# Risk of hospitalization and vaccine effectiveness among COVID-19 patients in the UAE during the Delta and Omicron outbreaks

**DOI:** 10.3389/fimmu.2023.1049393

**Published:** 2023-02-13

**Authors:** Mohammed Albreiki, Mira Mousa, Syafiq Kamarul Azman, Hema Vurivi, Zainab Alhalwachi, Fatima Alshehhi, Safiya AlShamsi, Nada Al Marzouqi, Tayba Alawadi, Hussain Alrand, Abderrahim Oulhaj, Asma Fikri, Habiba Alsafar

**Affiliations:** ^1^ Center for Biotechnology, Khalifa University of Science and Technology, Abu Dhabi, United Arab Emirates; ^2^ Nuffield Department of Women’s and Reproduction Health, Oxford University, Oxford, United Kingdom; ^3^ Department of Electrical Engineering and Computer Science, Khalifa University of Science and Technology, Abu Dhabi, United Arab Emirates; ^4^ Public Health Sector, Ministry of Health and Prevention, Dubai, United Arab Emirates; ^5^ National Center for Health Research, Ministry of Health and Prevention, Dubai, United Arab Emirates; ^6^ College of Medicine and Health Sciences, Khalifa University, Abu Dhabi, United Arab Emirates; ^7^ Research and Data Intelligence Support Center, Khalifa University, Abu Dhabi, United Arab Emirates; ^8^ Faculty of Medicine, University of Sharjah, Sharjah, United Arab Emirates; ^9^ Emirates Health Services Establishment, Dubai, United Arab Emirates; ^10^ Department of Biomedical Engineering, College of Engineering, Khalifa University of Science and Technology, Abu Dhabi, United Arab Emirates; ^11^ Department of Genetics and Molecular Biology, College of Medicine and Health Sciences, Khalifa University of Science and Technology, Abu Dhabi, United Arab Emirates; ^12^ Emirates Bio-Research Center, Ministry of Interior, Abu Dhabi, United Arab Emirates

**Keywords:** COVID-19, SARS-CoV-2, hospitalization, Delta, Omicron, UAE, vaccine effectiveness

## Abstract

**Introduction:**

A rapid increase in COVID-19 cases due to the spread of the Delta and Omicron variants in vaccinated populations has raised concerns about the hospitalization risk associated with, and the effectiveness of, COVID-19 vaccines.

**Method:**

This case–control study aims to determine the hospitalization risk associated with the inactivated BBIBP-CorV (Sinopharm) and mRNA BNT162b2 (Pfizer–BionTech) vaccines, and their effectiveness reducing the rate of hospital admission between 28 May 2021 and 13 January 2022, during the Delta and Omicron outbreaks. The estimation of vaccine effectiveness of 4,618 samples was based on the number of patients hospitalized at different vaccination statuses, adjusted for confounding variables.

**Results:**

Hospitalization risk increases in patients affected with the Omicron variant if patients are aged ≤ 18 years (OR 6.41, 95% CI 2.90 to 14.17; p < 0.001), and in patients affected with the Delta variant if they are aged > 45 years (OR 3.41, 95% CI 2.21 to 5.50; p < 0.001). Vaccine effectiveness reducing the rate of hospital admission for fully vaccinated participants infected with the Delta and Omicron variants was similar for both the BBIBP-CorV (94%, 95% CI 90% to 97%; 90%, 95% CI 74% to 96%) and BNT162b2 vaccines (95%, 95% CI 61% to 99.3%; 94%, 95% CI 53% to 99%), respectively.

**Discussion:**

The BBIBP-CorV and BNT162b2 vaccines utilized in the UAE vaccination program were highly effective in reducing the rate of COVID-19-related hospitalization during the Delta and Omicron outbreaks, and further effort must be taken to achieve high vaccine coverage rates in children and adolescents in the global context to reduce the hospitalization risk associated with COVID-19 on an international scale.

## Introduction

Coronavirus disease 2019 (COVID-19), caused by severe acute respiratory syndrome coronavirus 2 (SARS-CoV-2), has resulted in a huge burden on the healthcare system (1, 2). Up to 23 August 2019, SARS-CoV-2 has afflicted > 600 million people globally, leading to an estimated 6.5 million deaths in 192 countries (3). Ineffective public health mitigation measures, as well as the high transmission rate of SARS-CoV-2, have given rise to specific SARS-CoV-2 variants characterized as variants of concern (VOCs), such as Delta and Omicron (4, 5). This has prompted the WHO to collaborate with national authorities and pharmaceutical companies to develop therapeutic alternatives and vaccination formulations. To date, there are nine COVID-19 vaccines that have been approved by the WHO, including (1): two RNA vaccines—Moderna (mRNA-1273) and Pfizer–BionTech (BNT162b2) (2); three non-replicating viral vectors—Janssen: Pharmaceutical Companies of Johnson & Johnson (Ad26.COV2.S), Oxford–AstraZeneca (chAdOx1 nCoV19), and Serum Institute of India: Covishield (Oxford–AstraZeneca formulation) (3); two protein subunits—NVX-CoV2373 and Novavax (4); and inactivated virus techniques, Sinopharm (BBIBP-CorV) and Sinovac (CoronaVac) (6). The development of various vaccine types using different vaccine platforms has been a major component of the global COVID-19 pandemic response plan.

Several studies have been conducted to assess different COVID-19 vaccination programs, particularly those utilizing the BNT162b2, chAdOx1 nCoV19, and mRNA-1273 vaccines. These vaccines were associated with preventing symptomatic illness and reducing the numbers of cases of hospitalization and death caused by COVID-19 (7, 8). Vaccination campaigns with an inactivated SARS-CoV-2 vaccine (CoronaVac) have been largely conducted in several countries with varying estimated efficacy against asymptomatic illness, with Brazil and Indonesia reporting 50.6% and 65.30% effectiveness, respectively, and Turkey, where a substantially higher efficacy in preventing hospitalization was observed, reporting 83.50% effectiveness (9–11). Recently, the CoronaVac vaccine has shown effectiveness in preventing hospitalization (87.5%), critical hospital admission (90.3%), and death (86.3%) in the Chilean population (12).

The UAE’s COVID-19 vaccination strategy has been world-leading. By the first quarter of 2021, the UAE government initiated a large vaccination program to vaccinate its residents against coronavirus. As of March 2022, over 24 million vaccine doses had been administered and 96% of the population had been fully vaccinated, ranking UAE among the top countries in the vaccination campaign (13, 14). There are five vaccines in the UAE that have been used to protect its population from COVID-19 infection: these are the Sinopharm (BBIBP-CorV), BNT162b2, Moderna, Oxford–AstraZeneca, and Sputnik V (Gam-COVID-Vac) vaccines. BBIBP-CorV and BNT162b2 have been the most widely used in the UAE vaccination campaign. The initiated Phase III clinical trials of BBIBP-CorV in the UAE have revealed a vaccine efficacy of 78.1% and 72.8% for the HB02 and WIV04 strains, respectively (15), whereas in a real-world scenario, in fully vaccinated individuals with BBIBP-CorV, a post-vaccine effectiveness of 80%, 92%, and 97% in preventing hospitalization, critical care admission, and death, respectively, has been shown (16).

Despite a large number of clinical trials and studies on vaccine effectiveness, the majority of studies were conducted before the emergence of the Delta and Omicron VOCs. VOCs have been shown to decrease antibody neutralization because they tend to facilitate such key antibody-neutralizing mutations as L452R, E484K/A, K417N, T478K, P681R, and S477N (17–19). In turn, this results in an increase in transmissibility, reinfection (20, 21), and risk of hospitalization (22, 23), particularly in unvaccinated or partially vaccinated patients (24). The advent of new variants has raised global public health concerns about the possible role of increased disease severity, immune escape, and antibody response. This study aims to determine the effectiveness of the BBIBP-CorV (Sinopharm) and BNT162b2 (Pfizer–BionTech) vaccines in reducing hospital admissions during the Delta and Omicron variant outbreaks. In addition, the present study assesses the impact of the Delta and Omicron variants on the severity of the patients (i.e., hospitalization outcomes) of the patient, and determines the impact of circulating mutations and the antibody-resistant mutations underlying the evolution of the Delta and Omicron variants on vaccination and severity of the patients.

## Methods

### Study design and population

We conducted an observational, case–control study in Dubai and the Northern Emirates (Sharjah, Ajman, Ras Alkhaimah, Umm AlQuwain, and Fujairah). Randomized community and intensive care unit facilities with targeted methods of sample collection of reverse transcription-polymerase chain reaction (RT-PCR) -positive specimens were adopted, following the European Centre for Disease Prevention and Control (ECDC)’s model to control misclassification and ascertainment bias (25). Samples were taken between 28 May 2021 and 13 January 2022. Participants were selected if they met the following inclusion criteria: had a positive COVID-19 test, was a resident of the UAE, and were able to provide informed consent and commit for the duration of the study. Samples were identified as being COVID-19 positive if they were diagnostically SARS-CoV-2 positive with amplification of the targeted *ORF*, *N*, and *S* genes, as outlined in WHO guidelines. Information about the study was provided to potential participants. Participant demographic characteristics, severity of the patients, and vaccination status were extracted from their electronic health records.

This study has received a favorable ethics opinion from the local ethics committee at the Ministry of Health and Prevention (MOHAP) (DXB-REC/AAA/No. 80/2021). Participant information was coded and held securely in compliance with the data protection regulations of Khalifa University. All study procedures were conducted in accordance with international ethics standards (e.g., Declaration of Helsinki, 1964) and UAE Federal Law No (4). of 2016.

### Severity and vaccine classification

Confirmed positive cases were classified as either non-hospitalized or hospitalized according to the national diagnosis and treatment protocol for COVID-19 in the UAE. Non-hospitalized patients were individuals with asymptomatic to mild clinical presentations, whose symptoms did not limit their engagement in their day-to-day activities. This comprised patients presenting with fever, cough, sore throat, malaise, headache, muscle pain, nausea, vomiting, diarrhea, and loss of taste and smell, but who did not have shortness of breath, dyspnea, or abnormal chest imaging. Patients in this category isolated either at home or in non-hospital isolation facilities. Hospitalized patients were those with moderate to critical symptoms, such that they required supervision or supportive care in a hospital setting, or who were at risk of developing severe disease. This category also included patients who presented with lower respiratory disease, respiratory failure, septic shock, or organ dysfunction during their clinical assessments, and who therefore required hospitalization or admission to the ICU department.

Data related to patients’ vaccination status, including the name of the vaccine, the number of doses, and vaccination date, were provided by MOHAP. Patients were classified as either unvaccinated, partially vaccinated, or fully vaccinated. Patients were considered unvaccinated if they had not received the COVID-19 vaccine, or had a positive RT-PCR test for SARS-CoV-2, before the onset of their symptoms. Partially vaccinated patients were those who had received their first vaccine dose between 1 and 14 days after a positive PCR test for SARS-CoV-2 (for cases in which patients received two vaccine doses). Fully vaccinated patients were those who had received two vaccine doses, or had a positive PCR test for SARS-CoV-2, at least 14 days before the onset of their symptoms.

### Library preparation phylogeny construction

COVID-based libraries were prepared for sequencing using the Illumina^®^ COVIDSeq™ Test and IDT^®^ for Illumina-PCR Dual Indexes Set 1–4 (CAT#: 20043137, San Diego, CA, USA). The prepared libraries were sequenced using NovaSeq 6000 SP reagent kit, v1.5 (100 cycles) (CAT#: 20028401, San Diego, CA, USA). Identification of the variants of concern was based on the in-house-developed CoVSeQ pipeline following the instructions recommended by the Broad Institute’s genome analysis tool kit (i.e., GATK) (26). Further information on the in-house CoVSeQ pipeline and phylogeny construction is detailed in **Supplementary Text 1**.

### Data quality and sample inclusion

**Supplementary Figure 1** illustrates the selection criteria applied in this study during the data filtering steps. Samples with missing demographic data (i.e., age, sex, nationality, patient status, vaccine status, and vaccine type) were removed from the subsequent analysis (*n* = 698). Samples that exhibited less than 90% of the reference-based mapping with at least a 10-fold coverage were excluded from this study (*n* = 335). Further quality checks were undertaken using Nextclade 0.14.1 default quality control analyses, that is those for missing data, mixed sites, private mutations, mutation clusters, stop codons, and frameshifts. Samples with bad quality scores (i.e., individual quality control score ≥ 100) were excluded from the subsequent analysis (*n* = 395) (27). Samples that failed the quality metrics of PANGOLIN (Phylogenetic Assignment of Named Global Outbreak Lineages) assigner (scorpio call, conflict, and ambiguity score) were removed from this study (*n* = 316) (28). In total, 758 samples were excluded from this study (**Supplementary Figure 1**). The final study population that passed filtration was 4,618.

### Statistical analysis

All data analyses were performed using Statistical Package for Social Science software (SPSS; IBM SPSS Statistics, IBM Corporation, Armonk, NY, USA) version 25 and GraphPad Prism version 7 (GraphPad Prism, San Diego, CA, USA). The descriptive variables were assessed using frequency analysis for categorical variables whereas mean and standard deviation were used for continuous measures. We have applied cross-tabulation to estimate the association between vaccine status and other variables (i.e., age, sex, patient status, variant of concern, and vaccine type). Multivariate logistic regression models were used to test the relationship between different variables (i.e., vaccine status vs. patient status; vaccine status vs. lineage type; patient status vs. lineage type; and mutation vs. patient status). All regression models were adjusted for age (continuous), nationality, and sex. We have used multivariate logistic regression analysis to estimate the odds ratio for hospitalization by comparing the odds of different vaccination statuses between non-hospitalized and hospitalized patient groups. Vaccine effectiveness (VE) was estimated using the following formula: vaccine effectiveness = (1 − odds ratio) × 100. Lastly, we estimated vaccine effectiveness stratified by age group (i.e., ≤ 44 years and ≥ 45 years) and VOCs (i.e., Delta and Omicron).

For mutation analysis, the cross-tabulation was conducted on all mutations to test the statistical significance at a *p-*value of <0.05 (**Supplementary Table 1**). Only mutations that were present in 10% of the samples were selected for mutation analysis. Multivariate logistic regression analysis was used to investigate the association between the most common mutations and the risk of hospitalization, adjusting for covariates such as age and sex. Pie charts were created to show the proportions of antibody-resistant variants among fully vaccinated and unvaccinated individuals. Fisher’s exact test (two-tailed) was used to determine the statistically significant differences in the distribution of antibody-resistant variants of Delta (the L452R, T478K, and P681R mutations) and Omicron (E484A, K417N, and S477N) among fully vaccinated patients (17–19). The results of all tests were considered statistically significant if the *p*-value > 0.05.

## Results

### Patient characteristics

The demographic, geographical, and clinical characteristics of 4,618 patients are shown in **Table 1**. The sex distribution of the study population was 51.5% male and 48.1% female. The largest proportion of cases was in patients who were aged between 19 and 44 years (48.4%), and from the Middle East and Asia (64.2% and 23.8%, respectively). The mean age was 51.6 ± 20.4 years in hospitalized patients, and 29 ± 17.1 years in non-hospitalized patients. The largest proportion of hospitalized and non-hospitalized patients was reported in those who were aged > 45 years and 19–44 years, respectively (*n* = 348, 64.3%; *n* = 2,068, 50.7%). The majority of patients who were non-hospitalized (88.3%) were diagnosed with the Delta variant (84.1%). Patients with an unvaccinated status represented the largest proportion of hospitalized cases (*n* = 382, 70.6%), whereas patients with a fully vaccinated status represented the largest proportion of non-hospitalized cases (*n* = 1,812, 44.4%). Excluding missing variables, the largest proportion of vaccine type administered was reported for the BBIBP-CorV and BNT162b2 vaccines (59.1% and 7.2%, respectively).

**Table 1:** Demographics and characteristics of study participants.

Factor	Non-hospitalized, *n* (%) [N = 4,077 (88.3%)]	Hospitalized, *n* (%) [N = 541 (11.7%)]	Total, *n* (%) N = 4,618
Age (mean ± SD)	29 ± 17.1	51.6 ± 20.4	32 ± 18.8
< 18 years	1,227 (30.1%)	27 (5.0%)	1,254 (27.2%)
19–44 years	2,068 (50.7%)	166 (30.7%)	2,234 (48.4%)
> 45 years	779 (19.1%)	348 (64.3%)	1,127 (24.4%)
Missing	3 (0.1%)	0 (0.0%)	3 (0.1%)
Sex, *n* (%)			
Male	2,085 (50.1%)	294 (54.3%)	2,379 (51.5%)
Female	1,990 (48.8%)	233 (43.1%)	2,223 (48.1%)
Missing	2 (0.04%)	14 (2.6%)	16 (0.3%)
Nationality, *n* (%)			
Middle Eastern	2,671 (65.5%)	293 (54.2%)	2,964 (64.2%)
Asian	901 (22.1%)	199 (36.8%)	1,100 (23.8%)
African	375 (9.2%)	45 (8.3%)	420 (9.1%)
European	26 (0.6%)	2 (0.4%)	28 (0.6%)
American	12 (0.3%)	2 (0.4%)	14 (0.3%)
Missing	92 (2.1%)	0 (0.0%)	92 (2.0%)
Vaccination status, *n* (%)			
Partially vaccinated	162 (4.0%)	45 (8.3%)	207 (4.5%)
Fully vaccinated	1,812 (44.4%)	85 (15.7%)	1,897 (41.1%)
Unvaccinated	1,317 (32.3%)	382 (70.6%)	1,699 (36.8%)
Missing	786 (19.3%)	29 (5.4%)	815 (17.6%)
Vaccine type, *n* (%)			
BBIBP-CorV	1,606 (58.1%)	120 (22.2%)	1,726 (37.4%)
BNT162b2	200 (7.2%)	8 (1.5%)	208 (4.5%)
Others^†^	32 (1.4%)	31 (5.7%)	32 (0.7%)
Missing	922 (33.4%)	382 (70.6%)	953 (20.6%)
Variant of concern, *n* (%)			
Delta	3,410 (83.6%)	475 (87.8%)	3885 (84.1%)
Omicron	667 (16.4%)	66 (12.2%)	733 (15.9%)

Missing variables were not included in subsequent analyses.

†This includes other vaccine types such as ChAdOx1, JNJ-78436735, and Gam-COVID-Vac.

### VOC distribution in the UAE

**Figure 1:**
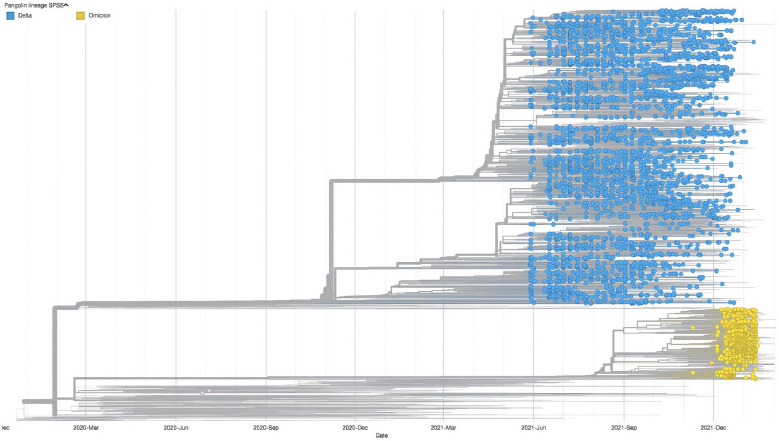
Timescale phylogentic tree of the SARS-CoV-2 VOCs (shown as circle) in the UAE from late May 2021 to the end of January 2022, contextualized with the 1,993 most smilar sequences selected from Global Initiative on Sharing Avian Influenza Data. VOCs, variants of concern.

A time-scaled phylogeny of 4,618 sequenced samples taken between May 2021 and January 2022 is presented in **Figure 1**. The predominance of the Delta variant (B.1.617.2; **Figure 1**, blue dot) and its sublineages is clearly seen between May 2021 and December 2021. We have identified approximately 100 sublineages of the Delta variant, with the AY.102 sublineage representing the highest proportion (26.9%). The heterogeneous distribution of the Delta sublineages indicates the presence of the major Delta clades (21J, 74%; 21K, 18%; 21I, 4%; and 21A, 3%). The first reported Omicron variant (B.1.1.529; **Figure 1**, yellow) was in November 2021, and by January 2022, the prevalence of the Omicron variant and its sublineages (BA.1, BA.1.1, and BA.2) was higher than that of the Delta variant.

### Hospitalization status and VOCs

**Table 2** demonstrates the association between patient hospitalization status and the presence of VOCs (i.e., Delta and Omicron), stratified by age groups (i.e., ≤ 45 years and > 45 years) and adjusted for sex. In the ≤ 45 years age group, there was no significant difference in hospitalization status for those patients infected with the Omicron (OR 1.31, 95% CI 0.90 to 1.87; *p* = 0.15) and Delta variants (OR 0.76, 95% CI 0.53 to 1.10; *p* = 0.15). Interestingly, after further stratification between three age groups (i.e., ≤ 18 years; 18–45 years; and > 45 years), we identified a significant association with hospitalization status for those in the ≤ 18 years age group; namely, that those infected with the Delta variant were less likely to be hospitalized (OR 0.15, 95% CI 0.07 to 0.34; *p* < 0.001). On the other hand, those infected with the Omicron variant were more likely to be hospitalized (OR 6.41, 95% CI 2.90 to 14.17; *p* < 0.001), as demonstrated in **Supplementary Table 1**. In the > 45 years age group, there was a significant difference in hospitalization status; namely, that those infected with the Delta variant were more likely to be hospitalized (OR 3.41, 95% CI 2.21 to 5.50; *p* < 0.001). Alternatively, those infected with the Omicron variant were less likely to be hospitalized (OR 0.29, 95% CI 0.18 to 0.47; *p* < 0.001).

**Table 2:** Patient status stratified by age group (i.e., ≤ 45 years and > 45 years), analyzed by the variant of concern (either the Delta or the Omicron variant) in association with hospitalization status (i.e., non-hospitalized and hospitalized).

Age group	Patient status	Frequency	Crude OR (95% CI)	*p*-value	Adjusted OR (95% CI)	*p*-value
≤ 45 years	**Delta** Non-hospitalized Hospitalized	2,773 (94.8%) 152 (5.2%)	1.00 0.69 (0.48 to0.99)	0.05	1.00 0.76 (0.53 to 1.10)	0.15
	**Omicron** Non-hospitalized Hospitalized	522 (92.7%) 41 (7.3%)	1.00 1.43 (1.00 to 2.04)	0.05	1.00 1.31 (0.90 to 1.87)	0.15
> 45 years	**Delta** Non-hospitalized Hospitalized	634 (66.2%) 323 (33.85%)	1.00 2.95 (1.89 to4.61)	< 0.001	1.00 3.41 (2.12 to 5.50)	< 0.001
	**Omicron** Non-hospitalized Hospitalized	145 (85.3%) 25 (14.7%)	1.00 0.33 (0.21 to 0.52)	< 0.001	1.00 0.29 (0.18 to 0.47)	< 0.001

CI, confidence interval; OR, odds ratio.

Reference categories: non-hospitalized.

Stratified by age groups ≤ 45 years, and > 45 years.

Chi-squared test of significance was used to measure associations between reference category and each category in the model.

Bivariate analysis (non-hospitalized vs. hospitalized) was used for the regression models, presented as crude OR and adjusted OR for nationality and sex.

### Vaccine effectiveness and VOCs

Overall, in comparison to the unvaccinated group of individuals, the vaccine effectiveness in those who were partially vaccinated was 54% (95% CI 28% to 71%); in those who were fully vaccinated, 97% (95% CI 96% to 98%); and in those who had received the third and fourth booster doses, 97% (95% CI 93% to 99%), as demonstrated in **Supplementary Table 2**. After stratification by age groups (i.e., the ≤ 45 years and > 45 years age groups), the effectiveness of all vaccine types against the Delta and Omicron variants was estimated, as shown in **Table 3**. With regard to the Delta variant, the vaccine effectiveness against hospital admission in fully vaccinated participants in the ≤ 45 years age group who had received the BBIBP-CorV and BNT162b2 vaccines was 94% (95% CI 90% to 97%) and 95% (95% CI 61% to 99.3%), respectively. Similarly, the vaccine effectiveness against hospital admission caused by COVID-19 in fully vaccinated participants in the > 45 years age group who had received the BBIBP-CorV and BNT162b2 vaccines was 92% (95% CI 88% to 95%) and 98% (95% CI 79% to 99.7%), respectively. The vaccine effectiveness in partially vaccinated participants in the > 45 years age group who had received the BNT162b2 vaccine was 82% (95% CI 20% to 96%), whereas no vaccine effectiveness was reported for partially vaccinated participants in the ≤ 45 years age group because of statistical insignificance (*p* = 0.51). Vaccine effectiveness in preventing hospitalization due to COVID-19 in participants in the > 45 years age group who had received the BBIBP-CorV booster vaccination was 94% (95% CI 49% to 99.3%).

**Table 3:** Estimated vaccine effectiveness against the variant of concern (either the Delta or Omicron variant), stratified by age group (≤ 45 years; > 45 years), and analyzed by type of vaccination (BBIBO-CoV) and vaccination status (BNT162b2).

Variant	Age group	Type of vaccination	Non-hospitalized	Hospitalized	Crude OR (95% CI)	*p-*value	Adjusted OR (95% CI)	*p-*value	Vaccine effectiveness (95% CI)
Delta	≤ 45 years	**BBIBP-CoV** Partially vaccinated Fully vaccinated	81 (8.0%) 936 (92%)	11 (42.3%) 15 (57.7%)	1.28 (0.58 to 2.30) 0.15 (0.09 to 0.27)	0.46 < 0.001	0.88 (0.43 to 1.79) 0.06 (0.03 to 0.10)	0.45 < 0.001	24% (–56% to 64%) 94% (90% to 97%)
		**BNT162b2** Partially vaccinated Fully vaccinated	26 (21.8%) 93 (78.2%)	1 (50%) 1 (50%)	0.36 (0.04 to 2.69) 0.10 (0.01 to 0.73)	0.32 0.02	0.24 (0.03 to 1.91) 0.05 (0.01 to 0.42)	0.18 0.01	76% (NA to 97%) 95% (58% to 99%)
	> 45 years	**BBIBP-CoV** Partially vaccinated Fully vaccinated	14 (4.3%) 314 (95.7%)	28 (38.9%) 44 (61.1%)	1.17 (0.59 to 2.30) 0.08 (0.05 to 0.12)	0.64 < 0.001	1.27 (0.62 to 2.65) 0.08 (0.05 to 0.12)	0.51 < 0.001	NA 92% (88% to 95%)
		**BNT162b2** Partially vaccinated Fully vaccinated	7 (25.9%) 20 (74.1%)	3 (75%) 1 (25%)	0.25 (0.06 to 0.98) 0.02 (0.0 to 0.22)	0.04 0.001	0.18 (0.04 to 0.80) 0.02 (0.004 to 0.22)	0.02 0.001	82% (20% to 96%) 98% (78% to 99.6%)
Omicron	≤ 45 years	**BBIBP-CoV** Partially vaccinated Fully vaccinated	4 (2.0%) 193 (98%)	0 (0.0%) 8 (100%)	NA 0.11 (0.05 to 0.26)	NA < 0.001	NA 0.10 (0.04 to 0.26)	NA < 0.001	NA 90% (74% to 96%)
		**BNT162b2** Partially vaccinated Full vaccinated	4 (8.2%) 45 (91.8%)	0 (0.0%) 1 (100%)	NA 0.06 (0.01 to 0.46)	NA 0.007	NA 0.06 (0.01 to 0.47)	NA 0.01	NA 94% (53% to 99%)
	> 45 years	**BBIBP-CoV** Partially vaccinated Full vaccination	2 (3.2%) 61 (96.8%)	1 (7.1%) 13 (92.9%)	0.60 (0.04 to 7.63) 0.25 (0.09 to 0.71)	0.69 0.01	0.37 (0.02 to 5.87) 0.42 (0.13 to 1.33)	0.48 0.14	63% (NA to 98%) 58% (NA to 87%)
		**BNT162b2** Partial Vaccination Full vaccination	0 (0.0%) 5 (100%)	0 (0.0%) 1 (100%)	NA 0.24 (0.02 to 2.41)	NA 0.22	NA 0.17 (0.01 to 2.23)	NA 0.18	NA 83% (NA to 99%)

CI, confidence interval; NA, not applicable; OR, odds ratio.

Reference categories: unvaccinated.

Stratified by lineage and the ≤ 45 years and > 45 years age groups.

Chi-squared test of significance was used to measure associations between reference category and each category in the model.

Bivariate analysis (non-hospitalized vs. hospitalized) was used for the regression models, presented as crude OR and adjusted OR for nationality and sex.

Vaccine effectiveness estimation was adjusted for the covariates, that is, nationality and sex, and calculated from the regression model [(1 − OR) * 100] and expressed as a percentage.

As for the Omicron variant, the vaccine effectiveness against hospital admission in fully vaccinated participants in the ≤ 45 years age group who had received the BBIBP-CorV and BNT162b2 vaccines was 90% (95% CI 74% to 96%) and 94% (95% CI 53% to 99%), respectively. In contrast, no vaccine effectiveness was reported for participants in the >45 years age group who had received the BBIBP-CoV and BNT162b2 vaccines because of statistical insignificance (*p* = 0.14 and *p* = 0.18, respectively). We reported no vaccination effectiveness against hospital admission in both the ≤45 years and >45 years age categories because of the small sample size of hospitalized cases (for partially vaccinated participants in the ≤45 years age group who had received the BBIBP-CorV and BNT162b2 vaccines, *n* = 0; for partially vaccinated participants in the >45 years who had received the BBIBP-CorV and BNT162b2 vaccines, *n* = 0 and *n* = 1, respectively).

### Mutation associated with hospitalized cases

Altogether, we identified 734 unique mutations affecting the protein amino acid sequence in both non-hospitalized and hospitalized patients. The largest proportion of mutations was found in the ORF1a region *(n* = 211), whereas the smallest proportion was found in the ORF7b region (*n* = 5). Only mutations that were present in 10% of the samples were selected for mutation analysis. The number of mutations based on the above criteria for non-hospitalized, and hospitalized patients was 34. Further investigations have been undertaken to assess the association between the antibody-resistant variants and patients’ vaccination status. We studied the distribution of antibody-resistant variants of Delta (L452R, T478K, and P681R) and Omicron (E484A, K417N, and S477N) among fully vaccinated patients (17–19).

Cross-tabulation across all mutations has shown that 18 mutations demonstrated significant relationship with patients’ hospitalization status (**Supplementary Table 3**). Mutations that showed significant association with hospitalization status (*n* = 18), were analyzed using multivariate logistic regression adjusting for covariates (i.e., age and sex) to evaluate mutations that are related to hospitalized cases (**Supplementary Table 4**). **Table 4** demonstrates that the presence of mutations, adjusted for age and sex, was likely to be greater in hospitalized than in non-hospitalized patients. We also reported that mutations in nucleocapsid protein (R203K, *p* < 0.001), ORF1a (T3255I, *p < *0.001), ORF3a (G49V, *p* < 0.001), and spike protein (D950N, *p* = 0.03; G142D, *p* < 0.001; L452R, *p* = 0.01; T478K, *p* = 0.01; T95I, *p* < 0.001) were associated with the risk of hospital admission.

**Table 4:** Mutations associated with hospitalized cases due to COVID-19.

	Frequency	Crude OR (95% CI)	*p-*value	Adjusted OR (95% CI)^†^	*p*-value
**N:R203K** **Non-hospitalized** **Hospitalized**	3406 (87.7%) 473 (12.2%)	1.00 0.53 (0.42,0.66)	< 0.001	1.00 0.61 (0.47,0.79)	< 0.001
**ORF1a:T3255I** **Non-hospitalized** **Hospitalized**	3731 (88.3%) 467 (11.1%)	1.00 0.58 (0.44,0.76)	< 0.001	1.00 0.61 (0.45,0.83)	< 0.001
**ORF3a:G49V** **Non-hospitalized** **Hospitalized**	402 (95.0%) 21 (5.0%)	1.00 0.36 (0.23,0.57)	< 0.001	1.00 0.40 (0.25,0.64)	< 0.001
**S:D950N** **Non-hospitalized** **Hospitalized**	33 (87.6%) 469 (12.4%)	1.00 1.52 (1.17,1.97)	< 0.001	1.00 1.41 (1.01,1.95)	0.03
**S:G142D** **Non-hospitalized** **Hospitalized**	569 (93.3%) 41 (6.7%)	1.00 0.50 (0.36,0.70)	< 0.001	1.00 0.42 (0.29,0.61)	< 0.001
**S:L452R** **Non-hospitalized** **Hospitalized**	3254 (87.5%) 465 (12.5%)	1.00 1.54 (1.20,1.99)	< 0.001	1.00 1.49 (1.08,2.07)	0.01
**S:T478K** **Non-hospitalized** **Hospitalized**	3901 (88.0%) 530 (12.0%)	1.00 2.17 (1.17,4.02)	0.01	1.00 2.54 (1.21,5.35)	0.01
**S:T95I** **Non-hospitalized** **Hospitalized**	2803 (89.6%) 326 (10.4%)	1.00 0.68 (0.57,0.82)	< 0.001	1.00 0.71 (0.57,0.87)	< 0.001

CI, confidence interval; NA, not applicable; OR, odds ratio.

†Odds ratios adjusted for age and sex.

### Predominance of antibody-resistant SARS-CoV2 variants in fully vaccinated participants

**Figure 2:**
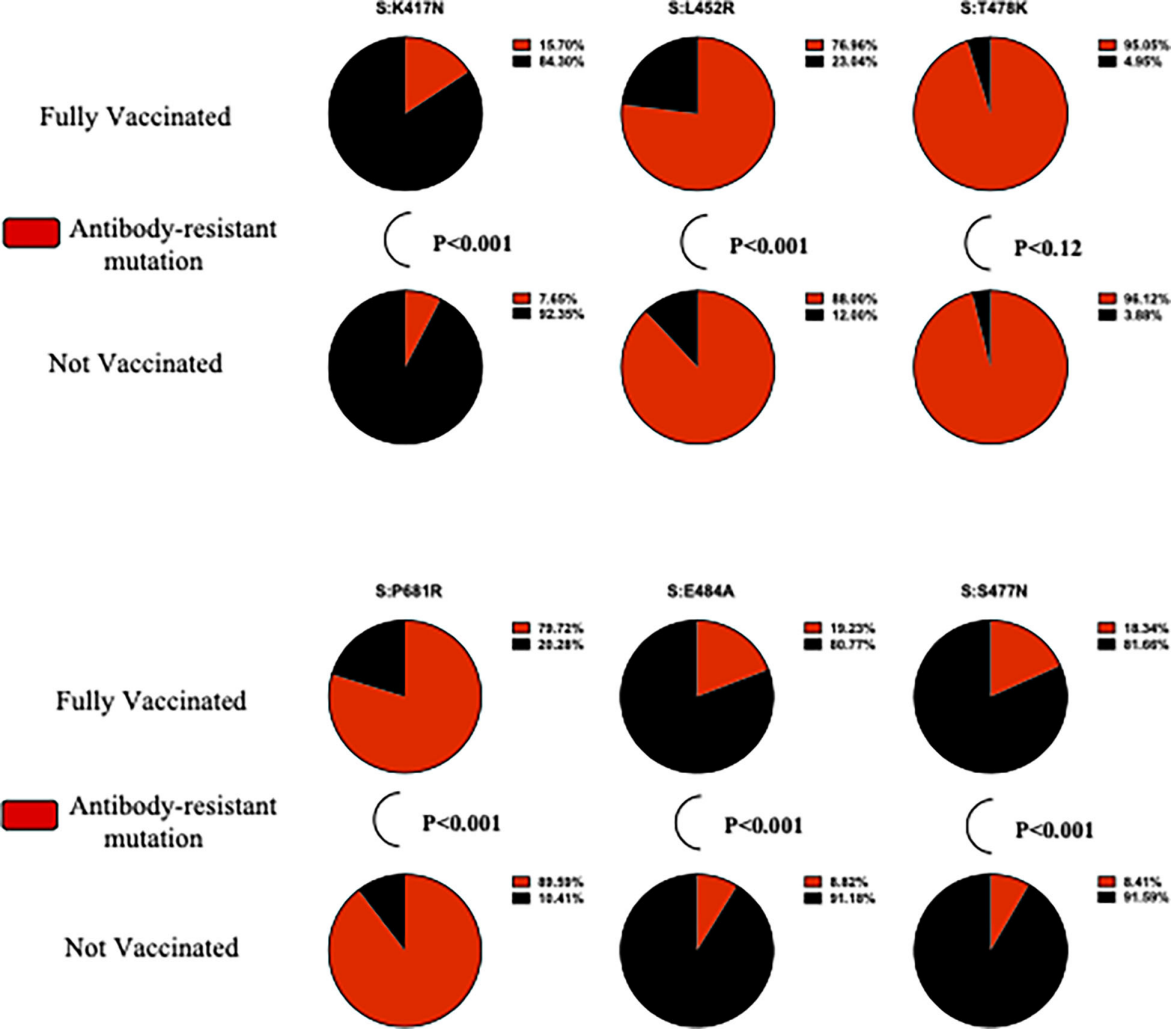
The proportion of SARS-CoV-2 genomes containing antibody-resistant variants.

**Figure 2** demonstrates the statistically significant differences in the proportion of antibody-resistant variants. S:K417N (*p < *0.001), S:E484A (*p < *0.001), and S:S477N (*p < *0.001) are more likely to be present in fully vaccinated participants, whereas S:L452R (*p < *0.001) and S:P681R (*p < *0.001) are more likely to be present in unvaccinated participants. No significant differences in the S:T478K proportion of fully vaccinated, compared with unvaccinated, participants were found (*p = *0.12). A complete illustration of antibody-resistant variants, including frequency and Fisher’s exact test, is presented in **Supplementary Table 5**.

## Discussion

In this study, the vaccine effectiveness of inactivated BBIBP-CorV and mRNA BNT162b2 vaccines against the risk of hospital admission was estimated for patients infected with the Delta and Omicron variants. The vaccine effectiveness in fully vaccinated participants infected with the Delta and Omicron variants against hospital admission was similar for participants who had received the BBIBP-CorV (Delta variant: 94%, 95% CI 90% to 97%; Omicron variant: 90%, 95% CI 74% to 96%) and the BNT162b2 vaccines (Delta variant: 95%, 95% CI: 61% to 99.3%; Omicron variant: 94%, 95% CI 53% to 99%), respectively. Hence, the BBIBP-CorV and BNT162b2 vaccines utilized in the UAE vaccination program were highly effective against COVID-19-related hospitalization during the Delta and Omicron outbreaks.

The analysis of 4,618 viral genomes collected in the UAE, indicates the presence of six major clades (21a, 21I, and 21 J for the Delta strain; and 21M, 21K, and 21L for the Omicron strain). Because of increase in travel by UAE residents, both internationally and within the UAE, the dramatic spread of the Delta variant in June 2021 was notable, 1 month after its emergence in Europe (29). Our data showed that it took 2 months for the VOC Delta strain and its sub-lineages to become dominant, indicating the steep increase in COVID-19 cases between May and November 2021, which can in turn be attributed to this variant’s high transmissibility and reduced sensitivity to the immune response (30). In November 2021, the VOC Omicron was first detected in the UAE, and became the dominant strain between December 2021 and January 2022, with a much higher reported prevalence than Delta in January 2022.

To estimate the age-specific susceptibility of COVID-19 related to hospitalization status, we stratified our analyses based on different age groups. Interestingly, in the ≤ 18 years age group, those infected with the Delta variant were less likely to be hospitalized (OR 0.15, 95% CI 0.07 to 0.34; *p* < 0.001). On the other hand, those infected with the Omicron variant were more likely to be hospitalized (OR 6.41, 95% CI 2.90 to 14.17; *p* < 0.001), demonstrating that there is a high risk of hospitalization among children and adolescents infected with the Omicron strain. This may be attributed to the limited WHO-approved vaccines available for this age group, which could explain why only 18.2% of child and adolescent participants were vaccinated, and in turn why children and adolescents may be more susceptible to contracting the Omicron infection than adults (31, 32). Omicron-driven primary infection and hospitalization could also be explained by increased infectivity (33), immune evasion (34), and a possible reduction in vaccine effectiveness. On the other hand, immune senescence (35), inflammation (36), cellular hyperfunction (37), and age-related epigenome changes may explain the increased risk of developing severe COVID-19 disease in older patients infected with the Delta strain (38). In the > 45 years age group, those affected with the Delta variant were more likely to be hospitalized (OR 3.41, 95% CI 2.21 to 5.50; *p < *0.001), whereas those affected with the Omicron variant were less likely to be hospitalized (OR 0.29, 95% CI 0.18 to 0.47; *p* < 0.001). The increased risk of hospitalization associated with the Delta variant was reported in multiple studies comparing Delta with other VOCs such as Alpha (39), Beta (40), Gamma (41), and Omicron (42, 43). In addition, our findings indicating the decrease in hospitalization risk and hospitalization-related care associated with the Omicron variant is in agreement with other studies (42, 43).

Overall, the vaccine effectiveness of those who were partially vaccinated was 54% (95% CI 28% to 71%), and 97% (95% CI 96% to 98%) for those who were fully vaccinated. During the Delta outbreak, the vaccine’s effectiveness against hospitalization risk in fully vaccinated individuals who had received the BIBBP-CorV vaccine in the ≤ 45 years and > 45 years age groups was 94% (95% CI 90% to 97%) and 92% (95% CI 88% to 95%), respectively, which is in line with other real-world studies (44, 45). For fully vaccinated individuals who had received the BNT162b2 vaccine who were ≤ 45 years of age and > 45 years of age, the vaccine effectiveness was 95% (95% CI 61% to 99.3%) and 98% (95% CI 79% to 99.7%), respectively. Our estimates of vaccine effectiveness in fully vaccinated individuals were higher than estimates reported in Scotland (83%) (46) and Israel (40.5%) (47) against symptomatic infections, as well as in the USA (88%) (48) and Qatar (93.4%) against severe outcomes. However, the similarity in vaccine effectiveness of inactivated vaccines and mRNA vaccines in this cohort demonstrates the importance of vaccination, irrespective of the vaccine type received, to protect against the Delta variant (49). Interestingly, partial vaccination with one dose of the BNT162b2 vaccine was effective (82%, 95% CI 20% to 96%) against hospitalization in patients aged > 45 years, which is a slightly higher effectiveness than the one-dose vaccine effectiveness reported in other studies, such as those carried out in Canada (76%), England (30%–33%), and Qatar (64%). The similarity in these estimates of vaccine effectiveness could be explained by adjustment of confounding variables, a similar interval between vaccine doses, sample size, and phenotypic status (50).

As for the Omicron variant, a similar vaccine effectiveness against the risk of hospitalization was reported in fully vaccinated participants in the ≤ 45 years age group who had received the BBIBP-CorV and BNT162b2 vaccines [90% (95% CI 74% to 96%) and 94% (95% CI 53% to 99%), respectively]. The effectiveness of the BNT162b2 vaccine against hospitalization during the global Omicron outbreak has been reported in the USA (86%) (51), South Africa (70%) (52), Scotland (68%) (53), and Denmark (55.2%). These studies were conducted using a test-negative design (52, 53), an *S* gene-positive status (53, 54), and a predominant circulating variant for unidentified samples (51). In our study, unidentified samples were excluded, yet adjustment for immunocompromised, underlying comorbidities, and vaccination time point was not conducted. The effectiveness of vaccines against the risk of hospitalization highlights the need for the massive rollout of vaccinations, particularly during the outbreak of a VOC, such as Omicron.

We have also conducted mutation analysis to identify the mutations that were associated with hospitalization. The defining mutations of the Delta (i.e., S:D950N and S:L452R) and Omicron variants (i.e., N:R203K, ORF1a:T3255I, S:G142D, S:T478K, and S:T95I) were significantly associated with an increased risk of hospitalization in our study population. In addition, ORF3a:G49V, which has been associated with increased stability of the ORF3a region, is also associated with an increased risk of hospitalization. Other mutations, such as N:R203K, S:L452R, S:D950N, S:T478K, and S:T95I, have been associated with increased viral loads (55), RNA expression (56), enhanced interaction of human angiotensin-converting enzymes 2 (ACE2) (57), and failures in host antibody immunization (58). Mutations, particularly in the spike region, affect the transmissibility, pathogenicity, and immune escape of SARS-CoV2 variants. The over-representation of antibody-resistant mutations in VOCs has raised major concerns regarding vaccine effectiveness against antibody-resistant SARS-CoV-2 variants, such as the Delta and Omicron variants. In this study, the proportion of antibody-resistant mutations was significantly greater in fully vaccinated than in unvaccinated participants, which is in accordance with the findings of Servellita et al. (2022) (19). The predominance of these reported antibody-resistant mutations has raised major concerns regarding the effectiveness of the UAE’s vaccination strategy against the spread of the Delta and Omicron variants.

It is important to note that our study has limitations, which in general, are inherent to any analysis of vaccine effectiveness. Owing to the lack of epidemiological data, including data related to participants’ travel habits, comorbidities, socioeconomic status, and phenotypic profile, our adjustment analysis of the confounding factors was impacted. In addition, our results were not adjusted for ethnicity, as the nationality given on participants’ passports was used as a surrogate for their ethnicity, and this was not always accurate. The effectiveness of vaccines against Omicron and its subvariants was not assessed in this study because of the limited sample size. Our mutation analysis may also be biased with regard to sampling, as only 11.7% of patients were classed as hospitalized, and the remaining patients were classed as non-hospitalized. The major strength of this study was the collection of vaccine details and severity status, with the associated classification of VOC. The classification of VOCs was based on whole-genome sequences in comparison to other approaches, such as the proxy measure of signature mutations [RT-qPCR (reverse transcription-quantitative polymerase chain reaction) variant genotyping]. In our study, vaccine effectiveness was determined when the Delta and Omicron strains were dominant strains worldwide, whereas other studies have determined vaccine effectiveness against the original Wuhan strain.

In conclusion, our study illustrates the epidemiological emergence of VOCs (i.e., Delta and Omicron) despite widespread vaccination, which in turn indicates the presence of antibody-resistant mutations. It also provides evidence of hospitalization risk being distinctly associated with the presence of VOCs. Our observational study demonstrated that the main available vaccines (i.e., BBIBP-CorV and BNT162b2) in the UAE were highly effective in the prevention of hospitalization during an intensive wave of the Delta and Omicron variants. Our results are largely consistent with Phase III trials and real-world retrospective studies (15, 16). This study can contribute to existing understanding of the global transmission of SARS-CoV-2 variants, as well as assessments of the COVID-19-related countermeasures and vaccination strategies adopted by the UAE government.

## Data availability statement

The data presented in the study are deposited in the GISAID repository, with the following accession number: EPI_ISL_16799633- EPI_ISL_16800130; EPI_ISL_16800155- EPI_ISL_16801057; EPI_ISL_16801179- EPI_ISL_16802108; EPI_ISL_16802203- EPI_ISL_16802889; EPI_ISL_16804896- EPI_ISL_16804909; EPI_ISL_16804911- EPI_ISL_16805668; EPI_ISL_16805670- EPI_ISL_16805825.

## Ethics statement

This study was approved by the local ethics committee at the Ministry of Health and Prevention (MOHAP/DXB-REC/AAA/No. 80/2021). Participants provided signed informed consent forms. Written informed consent to participate in this study was provided by the participants’ legal guardian/next of kin.

## Author contributions

AF and HA conceived the project to study the role of the virus and host in COVID-19 in the UAE to allow for a multicentered approach to study the contribution of the SARS-CoV-2 virus and its human host to the COVID-19 disease in the UAE. MM, MA, AF, and HA conceived the central research questions. HA, FA, SA, NM, TA, and AF defined the sampling strategy, managed the sample collection and preparation process from consenting patients, and were responsible for RNA extraction and delivery of samples to the laboratory. MA built the bioinformatic pipeline for variant calling and genomic epidemiology, and developed code for data preprocessing. MA analyzed the results and initiated the first draft of the manuscript. All authors on the primary list contributed to the data interpretation, critically reviewed the manuscript, and approved the final manuscript for submission.
