# Supplementary material for: Risk of hospitalization and vaccine effectiveness among COVID-19 patients in the UAE during the Delta and Omicron outbreaks
Source: Front Immunol. 2023 Feb 13;14:1049393. doi: 10.3389/fimmu.2023.1049393 (PMC9969353; doi:10.3389/fimmu.2023.1049393)
Supplement: Supplementary file 1 [file DataSheet_1.doc]

**Supplementary Files**

Number of individuals recruited: 6,635

**Identification**

Individuals excluded*: n = 1,046

Individuals included: n = 5,319

**Screening**

Individuals included: n = 4,618

Individuals excluded**: n = 698

**Included**

Total individuals: n = 4,618

Non-Hospitalized: n = 4,077

Hospitalized: n = 541

*Excluded due to failing the coverage cut-off, Nextclade and pangolin assigner metrics.

**Excluded due to missing vaccine status, and vaccine type data.

**Supplementary Figure 1. Flow chart of inclusion and exclusion criteria.**

**Supplementary Table 1. Patient status stratified by variant of concerns**

|  | Patient Status | Frequency | Crude OR  (95% CI) | *p* | Adjusted OR  (95% CI) | *p* |
| --- | --- | --- | --- | --- | --- | --- |
| <18 Years | **Delta**  Non-Hospitalized  Hospitalized | 1078 (98.6%)  15 (1.4%) | 1.00  0.17(0.07,0.37) | <0.001 | 1.00  0.15(0.07,0.34) | <0.001 |
|  | **Omicron**  Non-Hospitalized  Hospitalized | 149 (92.5%)  12 (7.5%) | 1.00  5.78(2.65,12.60) | <0.001 | 1.00  6.41(2.90,14.17) | <0.001 |
| 19-44 Years | **Delta**  Non-Hospitalized  Hospitalized | 1695 (92.5%)  15 (1.4%) | 1.00  1.04(0.68,1.57) | 0.85 | 1.00  1.01(0.66,1.54) | 0.94 |
|  | **Omicron**  Non-Hospitalized  Hospitalized | 373 (92.8%)  29 (7.2%) | 1.00  0.96(0.63,1.45) | 0.85 | 1.00  0.98(0.64,1.49) | 0.94 |
| >45 Years | **Delta**  Non-Hospitalized  Hospitalized | 634 (66.2%)  323 (33.85%) | 1.00  2.95(1.89,4.61) | <0.001 | 1.00  3.41(2.12,5.50) | <0.001 |
|  | **Omicron**  Non-Hospitalized  Hospitalized | 145 (85.3%)  25 (14.7%) | 1.00  0.33 (0.21,0.52) | <0.001 | 1.00  0.29(0.18,0.47) | <0.001 |
| CI, Confidence Interval; OR, Odds Ratio  Reference categories: Non-Hospitalized  Stratified by age groups <18 years, 19-44 years and >45 years  Chi-squared test of significance was used to measure associations between reference category and each category in the model.  Bivariate analysis (non-hospitalized vs hospitalized) was used for the regression models, presented as crude OR and adjusted OR for age, nationality and gender | | | | | | |

**Supplemental Table 2. Vaccine Effectiveness of the cohort**.

|  | Crude OR (95% CI) | *P* value | Adjusted OR (95% CI) | *P* Value | Vaccine Effectiveness (95% CI) |
| --- | --- | --- | --- | --- | --- |
| Unvaccinated  Partially Vaccinated | 1.00  0.95(0.67,1.35) | 0.8 | 1.00  0.46(0.29,0.72) | 0.001 | 54%(28%, 71%) |
| Unvaccinated  Fully Vaccinated | 1.00  0.16(0.12,0.20) | <0.001 | 1.00  0.03(0.02,0.04) | <0.001 | 97%(96%,98%) |
| Unvaccinated  Booster Dose | 1.00  0.19(0.08,0.41) | <0.001 | 1.00  0.03(0.01,0.07) | <0.001 | 97%(93%,99%) |
| Bivariate analysis (non-hospitalized vs hospitalized) was used for the regression models, presented as crude OR and adjusted OR for age, nationality and gender | | | | | |

**Supplementary Table 3. Descriptive statistics of the SARS-CoV-2 variants with the patient status (non-hospitalized vs. hospitalized)**

| Mutation |  | Non-Hospitalized | Hospitalized | Pearson Chi-square | *P* value |
| --- | --- | --- | --- | --- | --- |
| M:I82T | No  Yes | 671 (90.8%)  3406 (87.8%) | 68 (9.2%)  473 (12.2%) | 5.37 | 0.02 |
| N:D377Y | No  Yes | 772 (90.3%)  3305 (87.8%) | 83 (9.7%)  458 (12.2%) | 4.08 | 0.04 |
| N:D63G | No  Yes | 674 (91.1%)  3403 (87.8%) | 66 (8.9%)  475 (12.2%) | 6.66 | 0.01 |
| N:G215C | No  Yes | 983 (87.6%)  3094 (88.5%) | 139 (12.4%)  402 (11.5%) | 0.65 | 0.42 |
| N:R203K | No  Yes | 2766 (86.5%)  1311 (92.3%) | 432 (13.5%)  109 (7.7%) | 32.34 | <0.001 |
| ORF1a:A1306S | No  Yes | 990 (87.8%)  3087 (88.4%) | 137 (12.2%)  404 (11.6%) | 0.28 | 0.59 |
| ORF1a:P2046L | No  Yes | 969 (87.8%)  3108 (88.4%) | 135 (12.2%)  406 (11.6%) | 0.37 | 0.54 |
| ORF1a:P2287S | No  Yes | 1244 (88.2%)  2833 (88.3%) | 167 (11.8%)  374 (11.7%) | 0.02 | 0.86 |
| ORF1a:T3255I | No  Yes | 346 (82.4%)  3731 (88.9%) | 74 (17.6%)  467 (11.1%) | 15.57 | <0.001 |
| ORF1a:T3646A | No  Yes | 994 (87.7%)  3083 (88.5%) | 140 (12.3%)  401 (11.5%) | 0.57 | 0.44 |
| ORF1a:V2930L | No  Yes | 992 (87.3%)  3085 (88.6%) | 144 (12.7%)  397 (11.4%) | 1.34 | 0.24 |
| ORF1b:A1918V | No  Yes | 980 (88.3%)  3097 (88.3%) | 130 (11.7%)  411 (11.7%) | 0.0 | 0.99 |
| ORF1b:G662S | No  Yes | 685 (90.7%)  3392 (87.8%) | 70 (9.3%  471 (12.2%) | 5.21 | 0.02 |
| ORF1b:P1000L | No  Yes | 688 (90.6%)  3389 (87.8%) | 71 (9.4%)  470 (12.2%) | 4.89 | 0.02 |
| ORF1b:P314L | No  Yes | 24 (96.0%)  4053 (88.2%) | 1 (4.0%)  540 (11.8%) | 1.44 | 0.22 |
| ORF3a:S26L | No  Yes | 676 (91.1%)  3401 (87.7%) | 66 (8.9%)  475 (12.3%) | 6.79 | <0.001 |
| ORF3a:G49V | No  Yes | 3675 (87.6%)  402 (95.0%) | 520 (12.4%)  21 (5.0%) | 20.51 | <0.001 |
| ORF7a:T120I | No  Yes | 871 (89.7%)  3206 (87.9%) | 100 (10.3%)  441 (12.1%) | 2.38 | 0.12 |
| ORF7a:V82A | No  Yes | 861 (90.0%)  3216 (87.8%) | 96 (10.0%)  445 (12.2%) | 3.3 | 0.06 |
| ORF7b:T40I | No  Yes | 829 (87.1%)  3248 (88.6%) | 123 (12.9%)  418 (11.4%) | 1.68 | 0.19 |
| ORF8:D119 | No  Yes | 719 (90.6%)  3358 (87.8%) | 75 (9.4%)  466 (12.2%) | 4.77 | 0.02 |
| ORF8:F120 | No  Yes | 722 (90.5%)  3355 (87.8%) | 76 (9.5%)  465 (12.2%) | 4.47 | 0.03 |
| ORF9b:T60A | No  Yes | 674 (91.1%)  3403 (87.8%) | 66 (8.9%)  475 (12.2%) | 6.66 | 0.01 |
| S:D614G | No  Yes | 59 (95.2%)  4018 (88.2%) | 3 (4.8%)  538 (11.8%) | 2.87 | 0.09 |
| S:D950N | No  Yes | 773 (91.5%)  33.04 (87.6%) | 72 (8.5%)  469 (12.4%) | 10.2 | <0.001 |
| S:E156 | No  Yes | 744 (90.0%)  3333 (87.9%) | 83 (10.0%)  458 (12.1%) | 2.74 | 0.09 |
| S:F157 | No  Yes | 744 (90.0%)  3333 (90.5%) | 83 (10.0%)  458 (12.1%) | 2.74 | 0.09 |
| S:G142D | No  Yes | 3508 (87.55%)  569 (93.35%) | 500 (12.55%)  41 (6.75%) | 16.94 | <0.001 |
| S:L452R | No  Yes | 823 (91.5%)  3254 (87.5%) | 76 (8.5%)  465 (12.5%) | 11.47 | 0.001 |
| S:P681R | No  Yes | 729 (91.2%)  3348 (87.7%) | 70 (8.8%)  471 (12.3%) | 8.15 | <0.001 |
| S:R158G | No  Yes | 745 (90.0%)  3332 (87.9%) | 83 (10.0%)  458 (12.1%) | 2.78 | 0.09 |
| S:T19R | No  Yes | 703 (89.7%)  3374 (88.0%) | 81 (10.3%)  460 (12.0%) | 1.74 | 0.18 |
| S:T478K | No  Yes | 176 (94.1%)  3901 (88.0%) | 11 (5.9%)  530 (12.0%) | 6.41 | 0.01 |
| S:T95I | No  Yes | 1274 (85.6%)  2803 (89.6%) | 215 (14.4%)  326 (10.4%) | 15.76 | <0.001 |

**Supplementary Table 4. Logistic regression of the SARS-CoV-2 variants with patient status (non-hospitalized vs. hospitalized).**

| Mutation | Patient Status | Crude OR  (95% CI) | *P* value | Adjusted OR (95% CI) | *P* value |
| --- | --- | --- | --- | --- | --- |
| M:I82T | Non-Hospitalized  Hospitalized | 1.00  1.37 (1.04-1.79) | 0.02 | 1.00  1.20 (0.85-1.70) | 0.28 |
| N:D377Y | Non-Hospitalized  Hospitalized | 1.00  1.28 (1.00-1.64) | 0.04 | 1.00  1.11 (0.86-1.51) | 0.5 |
| N:D63G | Non-Hospitalized  Hospitalized | 1.00  1.42 (1.08-1.86) | 0.01 | 1.00  1.28 (0.90-1.82) | 0.16 |
| N:G215C | Non-Hospitalized  Hospitalized | 1.00  0.91 (0.74-1.12) | 0.42 | 1.00  0.81 (0.63-1.03) | 0.09 |
| N:R203K | Non-Hospitalized  Hospitalized | 1.00  0.53 (0.42-0.66) | <0.001 | 1.00  0.61 (0.47-0.79) | <0.001 |
| ORF1a:A1306S | Non-Hospitalized  Hospitalized | 1.00  0.94 (0.76-1.16) | 0.59 | 1.00  0.82 (0.64-1.04) | 0.11 |
| ORF1a:P2046L | Non-Hospitalized  Hospitalized | 1.00  0.93 (0.76-1.15) | 0.54 | 1.00  0.80 (0.631-1.03) | 0.09 |
| ORF1a:P2287S | Non-Hospitalized  Hospitalized | 1.00  0.98 (0.81-1.19) | 0.86 | 1.00  0.87 (0.69-1.09) | 0.24 |
| ORF1a:T3255I | Non-Hospitalized  Hospitalized | 1.00  0.58 (0.44-0.76) | <0.001 | 1.00  0.61 (0.45-0.83) | <0.001 |
| ORF1a:T3646A | Non-Hospitalized  Hospitalized | 1.00  0.92 (0.75-1.13) | 0.44 | 1.00  0.80 (0.63-1.03) | 0.08 |
| ORF1a:V2930L | Non-Hospitalized  Hospitalized | 1.00  0.88 (0.72-1.08) | 0.24 | 1.00  0.78 (0.61-1.00) | 0.05 |
| ORF1b:A1918V | Non-Hospitalized  Hospitalized | 1.00  1.00 (0.811-1.23) | 0.99 | 1.00  0.87 (0.68-1.12) | 0.3 |
| ORF1b:G662S | Non-Hospitalized  Hospitalized | 1.00  1.35 (1.04-1.77) | 0.02 | 1.00  1.16 (0.83-1.63) | 0.36 |
| ORF1b:P1000L | Non-Hospitalized  Hospitalized | 1.00  1.34 (1.03-1.74) | 0.02 | 1.00  1.17 (0.84-1.65) | 0.3 |
| ORF1b:P314L | Non-Hospitalized  Hospitalized | 1.00  3.19 (0.43-23.68) | 0.25 | 1.00  2.48 (0.311-19.76) | 0.39 |
| ORF3a:S26L | Non-Hospitalized  Hospitalized | 1.00  1.43 (1.09-1.87) | <0.001 | 1.00  1.27 (0.89-1.81) | 0.17 |
| ORF3a:G49V | Non-Hospitalized  Hospitalized | 1.00  0.36 (0.23-0.57) | <0.001 | 1.00  0.40 (0.25-0.64) | <0.001 |
| ORF7a:T120I | Non-Hospitalized  Hospitalized | 1.00  1.19 (0.95-1.50) | 0.12 | 1.00  1.07 (0.81-1.41) | 0.63 |
| ORF7a:V82A | Non-Hospitalized  Hospitalized | 1.00  1.24 (0.98-1.56) | 0.06 | 1.00  1.10 (0.83-1.47) | 0.48 |
| ORF7b:T40I | Non-Hospitalized  Hospitalized | 1.00  0.86 (0.70-1.07) | 0.19 | 1.00  0.72 (0.56-0.93) | 0.01 |
| ORF8:D119 | Non-Hospitalized  Hospitalized | 1.00  1.33 (1.02-1.72) | 0.02 | 1.00  1.14 (0.83-1.59) | 0.4 |
| ORF8:F120 | Non-Hospitalized  Hospitalized | 1.00  1.31 (1.02-1.70) | 0.03 | 1.00  1.14 (0.82-1.57) | 0.42 |
| ORF9b:T60A | Non-Hospitalized  Hospitalized | 1.00  1.42 (1.08-1.86) | 0.01 | 1.00  1.26 (0.89-1.79) | 0.18 |
| S:D614G | Non-Hospitalized  Hospitalized | 1.00  2.63 (0.82-8.42) | 0.1 | 1.00  4.20 (1.00-18.40) | 0.05 |
| S:D950N | Non-Hospitalized  Hospitalized | 1.00  1.52 (1.17-1.97) | <0.001 | 1.00  1.41 (1.01-1.95) | 0.03 |
| S:E156 | Non-Hospitalized  Hospitalized | 1.00  1.23 (0.96-1.57) | 0.09 | 1.00  1.13 (0.83-1.55) | 0.41 |
| S:F157 | Non-Hospitalized  Hospitalized | 1.00  1.23 (0.96-1.57) | 0.09 | 1.00  1.13 (0.83-1.55) | 0.41 |
| S:G142D | Non-Hospitalized  Hospitalized | 1.00  0.50 (0.36-0.70) | <0.001 | 1.00  0.42 (0.29-0.61) | <0.001 |
| S:L452R | Non-Hospitalized  Hospitalized | 1.00  1.54 (1.20-1.99) | 0.001 | 1.00  1.49 (1.08-2.07) | 0.01 |
| S:P681R | Non-Hospitalized  Hospitalized | 1.00  1.46 (1.12-1.90) | <0.001 | 1.00  1.38 (0.98-1.95) | 0.05 |
| S:R158G | Non-Hospitalized  Hospitalized | 1.00  1.23 (0.96-1.57) | 0.09 | 1.00  1.14 (0.84-1.56) | 0.38 |
| S:T19R | Non-Hospitalized  Hospitalized | 1.00  1.18 (0.92-1.51) | 0.18 | 1.00  1.01 (0.73-1.39) | 0.92 |
| S:T478K | Non-Hospitalized  Hospitalized | 1.00  2.17 (1.17-4.02) | 0.01 | 1.00  2.54 (1.21-5.35) | 0.01 |
| S:T95I | Non-Hospitalized  Hospitalized | 1.00  0.68 (0.57-0.82) | <0.001 | 1.00  0.71 (0.57-0.87) | <0.001 |
| Bivariate analysis (non-hospitalized vs hospitalized) was used for the regression models, presented as crude OR and adjusted OR for age, nationality and gender | | | | | |

**Supplementary Table 5. Descriptive statistic and distribution of antibody-resistant variants among unvaccinated and fully vaccinated individuals.**

| Mutation | Fully Vaccinated | Unvaccinated | *P* value* |
| --- | --- | --- | --- |
| S:L452R  No  Yes | 435 (68.1%)  1463 (49.4%) | 204 (31.9%)  1496 (50.6%) | <0.001 |
| S:T478K  No  Yes | 94 (58.8%)  1804 (52.5%) | 66 (41.3%)  1634 (47.5%) | 0.12 |
| S:P681R  No  Yes | 385 (68.5%)  1513 (49.8%) | 177 (31.5%)  1523 (50.2%) | <0.001 |
| S:E484A  No  Yes | 1533 (49.7%)  365 (70.9%) | 1550 (50.3%)  150 (29.1%) | <0.001 |
| S:S477N  No  Yes | 1550 (49.9%)  348 (70.9%) | 1557 (50.1%)  143 (29.1%) | <0.001 |
| S:K417N  No  Yes | 1600 (50%)  298 (69.65) | 1570 (49.5%)  130 (30.4%) | <0.001 |
| *Fisher’s Exact Test | | | |

**Supplementary Text 1. In-house CovSeq Pipeline and Phylogeny construction**

In-house CovSeq pipeline was utilized in this study following the instructions recommended by the Broad Institute’s Genome Analysis ToolKit (GATK)^1^. The generated CovSeq reads were checked for quality using FastQC software (version 0.11.5)^2^. low quality reads (<Q30 ) and the presence of Illumina adapters were removed by Trimmmatric tool version 0.11.5^3^. Burrows-Wheeler Aligner (BWA – v.0.7.12) was used to map the trimmed reads to Wuhan-Hu-1-NC_045512.2/MN908947.3 reference genome^4^. The coverage for mapped reads were assessed using Qualimap (v2.2.1), indicating at least 90% of the reference based mapping exhibited at least 10-fold coverage^5^. Duplicated reads were removed by Picard tool (v.2.9.4) and the variant calling was determined using GATK Haplotype Caller, using a ploidy setting of 1 to account for the haploid genome of the virus. The generated FASTA of each samples were assigned for particular lineages using Pangolin COVID-19 lineage assigner (v 3.1.19)^6^. Also, clade assignment, mutation calling, and sequence quality checks were determined for each FASTA using Nextclade v1.13.2^7^. In addition, negative controls were applied in each run to ensure the absence of any contamination during the library preparation. For phylogentic tree generation, we have used a total of 4,618 sequences (filtered sequences as shown below) and compare them to the 3,060 SARS-CoV-2 GSAID sequences using Nucleotide-Nucleotide BLAST 2.6.0 (blastn)^8^.

^1^DePristo, M.A., et al., *A framework for variation discovery and genotyping using next-generation DNA sequencing data.* Nature genetics, 2011. **43**(5): p. 491-498.

^2^Andrews, S., *FastQC: a quality control tool for high throughput sequence data. 2010*. 2017.

^3^Bolger, A.M., M. Lohse, and B. Usadel, *Trimmomatic: a flexible trimmer for Illumina sequence data.* Bioinformatics, 2014. **30**(15): p. 2114-2120.

^4^Li, H., *Aligning sequence reads, clone sequences and assembly contigs with BWA-MEM.* arXiv preprint arXiv:1303.3997, 2013.

^5^McKenna, A., et al., *The Genome Analysis Toolkit: a MapReduce framework for analyzing next-generation DNA sequencing data.* Genome research, 2010. **20**(9): p. 1297-1303.

^6^O’Toole, Á., et al., *Assignment of epidemiological lineages in an emerging pandemic using the pangolin tool.* Virus Evolution, 2021. **7**(2): p. veab064.

^7^Hadfield, J., et al., *Nextstrain: real-time tracking of pathogen evolution.* Bioinformatics, 2018. **34**(23): p. 4121-4123.

^8^Altschul, S.F., et al., *Basic local alignment search tool.* Journal of molecular biology, 1990. **215**(3): p. 403-410.
